# “They Don’t See Us”: Asian Students’ Perceptions of Sexual Violence and Sexual Harassment on Three California Public University Campuses

**DOI:** 10.1177/08862605241235912

**Published:** 2024-03-12

**Authors:** Jianchao Lai, Eunhee Park, Claire Jo’Al Amabile, Sabrina C. Boyce, Rebecca Fielding-Miller, Dallas Swendeman, Laury Oaks, Daphne Marvel, Araz Majnoonian, Jay Silverman, Jennifer Wagman

**Affiliations:** 1 University of California Los Angeles, USA; 2 University of California San Diego, La Jolla, USA; 3 University of California Santa Barbara, USA; 4 San Diego State University, CA, USA

**Keywords:** Asians, college students, sexual violence, sexual harassment

## Abstract

Sexual violence and sexual harassment (SVSH) are prevalent among college and university students; however, the experiences of ethnic minority students, especially Asians, are understudied. This study aimed to reduce this gap by exploring Asian students’ perceptions of SVSH on three public university campuses in Southern California. We examined their perceptions about the campus environment related to SVSH, attitudes, and behaviors toward help seeking, and utilization of on-campus resources. A total of 23 in-depth interviews were conducted with Asian students enrolled at the three University of California campuses. Thematic coding was conducted to generate main themes and subthemes. Five main themes emerged: (a) SVSH is considered a “taboo” topic in Asian culture and family systems, and Asian student survivors are often reluctant to disclose incidents or seek support services. (b) Students did not feel their campus environments were tailored to understand or meet the sociocultural realities and needs of Asian student survivors. (c) Campus SVSH services and reporting processes were seen as non-transparent. (d) Peers were the major source of support and SVSH information, as opposed to official campus-based resources and training. (e) Survivors often conduct an internal cost–benefit analysis evaluating their decision about whether to report. This study highlights the lack of conversation surrounding SVSH in Asian families, and how the cultural stigma of sex and sexual violence prevented Asian students from receiving knowledge and resources about these topics in their families. Instead of relying on formal campus resources (e.g., Title IX and confidential advocacy services, mental health services), many students turn to their peers for support. Thus, facilitating peer support groups, training university students to support each other through SVSH incidents, and tailoring campus services to the diverse cultural backgrounds of students are key considerations to foster a safe campus environment and prevent SVSH.

## Introduction

Sexual violence is an overarching term used to represent a sexual act that is committed or attempted by a person without freely given consent of the victim or survivor or against someone unable to consent (Basile et al., 2014). Sexual harassment refers to sexual violations (i.e., unwelcome and inappropriate sexual remarks or physical advances) that occur in the workplace and/or a school setting (Hill & Kearl, 2011). Among college and university populations in the United States (U.S.), it is estimated that 20%–25% of students who identify as women experience sexual violence and/or sexual harassment (SVSH) victimization at some point during their time as a student (Conley et al., 2017; Fedina et al., 2018; Mellins et al., 2017; Voth Schrag, 2017). SVSH is associated with negative short- and long-term physical and mental health outcomes, including physical injury, reoccurring gynecological and sexual health problems, depression, anxiety, suicidal ideation, and symptoms of post-traumatic stress disorder (Basile et al., 2014; Stoner & Cramer, 2019).

Title IX of the Education Amendments of 1972 (Title IX) prohibits discrimination based on sex, and it provides a regulatory foundation to protect students from SVSH at institutions of higher education (U.S. Department of Education, 2008). Title IX requires that universities provide students who are victimized by SVSH with a mechanism to report the incident and receive accommodations to ensure their rights to receive education are upheld (Harper et al., 2017). However, while Title IX provides essential mechanisms for SVSH victim protection, it sometimes clashes with the constitutional principle of due process, which aims to safeguard the rights of students accused of perpetrating sexual violence, harassment, or misconduct (Harper et al., 2017). In addition, bureaucratic regulations and ineffective implementation of Title IX procedures can yield outcomes that fail to prioritize the safety and needs of the victims (Goldman, 2019). In general, college students encounter multiple challenges in reporting SVSH, including feelings of shame, privacy concerns, a lack of trust in the criminal justice system, and the fear of retaliation from the perpetrator (Griffin et al., 2022; Lathan et al., 2023; Mennicke et al., 2021; Sable et al., 2006; Schwarz et al., 2017; Zinzow & Thompson, 2011). In particular, Title IX has been found to present specific challenges for minoritized student groups, such as international students, including lack of knowledge about their rights and resources available to them, fear of visa status implications after officially reporting SVSH, limited access to support networks, cultural barriers, and stigma surrounding coming forward as a victim (Brubaker et al., 2017; Daigle et al., 2018; Holland & Cortina, 2017; Postel, 2015, 2020, Scholl et al., 2021).

Students exposed to SVSH commonly experience shame, guilt, embarrassment, and a desire to keep their victimization hidden from friends and family. These are significant factors that prevent victim-survivors from reporting SVSH incidents and seeking assistance afterward (Sabina & Ho, 2014). According to the Association of American Universities survey, only 15% of SVSH victims contact university agencies for help, most often due to the belief that the incident would not be taken seriously enough to merit any further action (Cantor et al., 2019). These attitudes and beliefs have largely been associated with cultural constructs that shape perceptions and attitudes toward SVSH, its disclosure, and willingness to report (Gilligan & Akhtar, 2006; Kenny & McEachern, 2000; Koo et al., 2012, 2015; Okazaki, 2002). Despite evidence about this understanding, there remains a notable gap in knowledge regarding the potential differences in SVSH-related reporting and help-seeking attitudes between different racial and ethnic student groups.

The Asian student and general Asian populations in the U.S.^1^ have been understudied in research focusing on violence and health (e.g., sexual violence, child abuse, suicide, mental health, and hate crimes) (Lantz & Wenger, 2022; Leong et al.,2007; Ninh, 2018; Zhai & Gao, 2009). Asians are often stereotypically portrayed as a “model minority” group who are perceived as being largely immune from social problems and characterized by high levels of education and income. This stereotype can elevate Asians to a status akin to “Honorary Whites” (Chanbonpin, 2015; Zhou, 2004; Wing, 2007). Internalization of the model minority myth can put the Asian population at risk for low service utilization, particularly in areas such as mental health, social support, and counseling. While Asian culture has demographic, cultural, and historical heterogeneity, Asians, compared to other ethnic groups, share similar cultural characteristics (Okazaki & Saw, 2011) that may serve as risk factors in the context of SVSH. For instance, studies have shown that parents convey messages like abstinence, gendered sex roles, and sex as taboo in Asian households. Such restrictive messages around sexuality reinforce conservative and gender-stereotypical attitudes (Kim & Ward, 2007; Kim, 2009; Liang et al., 2008; Malik et al., 2020; Morgan et al., 2010; Trinh & Kim, 2021). In a survey conducted with East and Southeast Asian college female students, respondents reported that their parents were the least common source of their sex education (Lee et al., 2013). Together, the model minority myth and traditional Asian cultural norms surrounding sex are potentially associated with the underreporting of SVSH among Asian college students (Lee & Law, 2001; Tjaden & Thoennes, 1998).

Intersectionality has become the predominant framework when conceptualizing complex systems of oppression that impact violence against women (Crenshaw, 1991). Social and institutional systems interact with racial and ethnic identities to produce distinct experiences of violence among women of racial minorities. The intersectional framework embraces contextual factors that are relevant to Asian American women with recent immigrant status and history, intergenerational trauma, and racialized sexism against Asian women (Buchanan et al., 2018). Asian American women have frequently been subjected to stereotypes like Japanese geishas and Filipina “mail-order brides,” resulting in sexual objectification and subservient images in the U.S. (Patel, 2008). It is likely that this stereotype, in turn, increases Asian American women’s risk for SVSH victimization and their reluctance to seek help.

Furthermore, experiences related to institutional betrayal are shown to exacerbate negative mental and physical health outcomes of SVSH on college and university campuses (Monteith et al., 2016; Rosenthal et al., 2016; Smith & Freyd, 2013). Institutional betrayal is defined as an institution’s problematic action or inaction about SVSH, ranging from isolated incidents to systemic failures (Smith & Freyd, 2014). Examples of institutional betrayal include covering up cases of campus-based SVSH, failing to take necessary prevention measures, and not providing effective and accessible services to survivors (Smith & Freyd, 2014). SVSH and institutional betrayal have disproportionally impacted racial and ethnic minority students, thus intersectionality and institutional betrayal together serve as the theoretical underpinnings of the current study (Cromer et al., 2018; Freyd & Birrell, 2013; Gómez, 2022).

This study presents findings from a qualitative exploratory study conducted to examine knowledge, attitudes, and experiences surrounding SVSH among Asian students across three University of California (UC) campus settings and explore how these results differ from those of the general student body. We present findings from 23 in-depth interviews (IDIs) conducted at three of the campuses in Southern California, to answer the following research questions: (a) What are Asian students’ perceptions of campus SVSH? and (b) What barriers and protective factors influence help-seeking behaviors among Asian college students?

The overarching goal of this study was to expand on the current scientific literature about the prevalence and impact of SVSH among college students in general, by lessening the gap that currently exists in the evidence on these issues among Asian students at U.S. institutions of higher education. Delving into the distinct experiences of Asian students, including both domestic and international students, is essential to uncovering the nuanced ways in which cultural norms, stereotypes, and institutional structures intersect to shape how students from this population understand and respond to SVSH. We share these findings to increase understanding of SVSH across diverse student populations and to contribute to the development of targeted interventions and support mechanisms that resonate with the specific needs and challenges faced by Asian students.

## Methods

### Research Design

This manuscript presents findings from a sub-analysis of data collected through the UC Speaks Up mixed-methods study conducted at UC Los Angeles (UCLA), UC San Diego (UCSD), and UC Santa Barbara (UCSB) (Wagman et al., 2022). These campuses represent a large public university school system, with undergraduate, graduate, and professional programs. Data were collected from January to June 2019 by a research team composed of seven faculty investigators, three staff members, and sixteen student researchers (11 undergraduates and 7 graduates). UC Speaks Up, the parent study, aimed to understand students’ perceptions of sexual violence on their UC campus, as well as their opinions of their campus’s quality, accessibility, and efficacy of existing on-campus prevention and intervention programming. This article specifically addresses the findings from student participants who self-identified as Asian.

### Research Participants and Procedures

We analyzed data from 23 IDIs conducted with Asian students across the three UC campuses. All 23 of these IDI participants were part of the UC Speaks Up “parent study” participant pool. Transcripts from students who self-identified as Asian on the study’s demographic form were selected for inclusion in the analytic sample. Both foreign- and U.S.-born Asian students were included. Each IDI typically lasted around 60–90 min, with an average interview time of 70 min. All IDIs were conducted in private spaces on one of the three campuses, such as in a library study room or a conference room. Undergraduate participants were matched with undergraduate student researchers, and graduate student participants were matched with a member of the graduate student research team. When possible, the researcher was paired with a participant of similar age and racial demographics. Student participants received a $25 Visa gift card to compensate them for their time.

The research team member conducting the IDI explained the purpose of the research study to each participant and allowed the opportunity for the participant(s) to ask any questions. The IDIs utilized semi-structured guides that allowed for organic dialog between the researchers and the participants. The guides included questions related to students’ perceptions of sexual consent within their community, sexual violence, and sexual harassment (i.e., the scale of the problem), reporting systems, survivor support efforts, existing violence prevention and interventions on their campuses, and recommendations for preventing sexual violence. Researchers used probes to elicit further information and to gain clarity about participants’ responses. All IDIs were recorded using a Sony hand-held audio recorder, with the consent of the participant(s).

### Study Recruitment

Participant recruitment was mainly done through convenience sampling strategies such as publicly displaying paper flyers to advertise the study across each campus and sharing electronic flyers through various listservs and social media accounts. Interested individuals were asked to complete an online form to screen for eligibility. The eligibility criteria included current enrollment at UCLA, UCSB, or UCSD; being over 18 years of age; having a way of being contacted (i.e., a cellphone number and/or email address), and being able to participate in an interview conducted in English. Eligible participants were contacted via email or cell phone by the research team after screening and invited to interview.

### Research Ethics

This study was approved by the UCSD Human Research Protection Program (HRPP) and the Institutional Review Boards at UCLA and UCSB gave reliance agreements that were linked to the UCSD HRPP approval. Before the data collection phase, the research team participated in a 3-day training on trauma-informed, survivor-centered, and ethical research practices, based on the guidelines for research on domestic violence against women by the World Health Organization (World Health Organization. Global Programme on Evidence for Health Policy, 2001). All researchers completed training provided by the Collaborative Institutional Training Initiative on Social-Behavioral-Educational research involving human subjects. The National Institutes of Health also supplied a Certificate of Confidentiality to protect sensitive information shared by the participants during interviews from mandated legal disclosure (e.g., experiences of sexual assault). Each IDI participant provided both verbal and written consent to be a part of the study and to allow audio recording of the interviews. To ensure consent was given ongoingly, the interviewers ensured that all participants were aware that they could terminate the interview at any point or skip any questions that made them feel uncomfortable with no incentive penalty.

### Data Analysis

All recordings of IDIs were transcribed verbatim into a Microsoft Word document by members of the research team. All word-processed transcriptions were thoroughly edited to remove any identifiable information, including names and affiliations (e.g., Greek organizations, departments, and employment). An encrypted, password-protected virtual folder was used to store all personal identifying information as well as the transcriptions. Only members of the research team had access to the encrypted folder.

**Figure 1.**
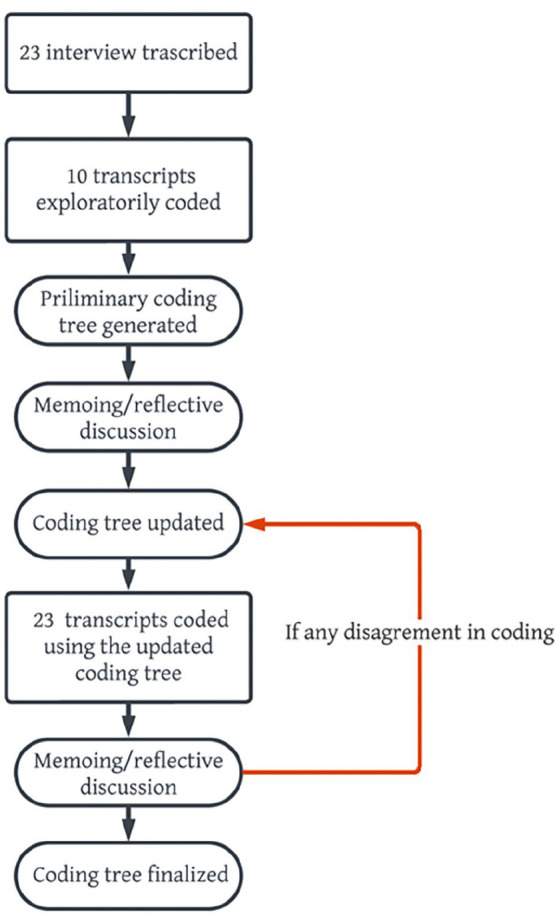
Coding process.

The analysis utilized a unique thematic coding method guided by Constructivist Grounded Theory techniques which are used for actively constructing knowledge by iterative coding, comparing, and summarizing findings in the data while recognizing the interpretive nature of the research (Braun & Clark, 2006; Charmaz, 2014). Specifically, the research team started open coding the first 10 transcripts line-by-line and developed the preliminary coding tree. Those codes captured the most basic and immediate meanings found within the data, focusing on the participants’ points of view and actions. Next, the team followed an iterative process of conducting team discussions, memoing, and cross-coding that generated an updated coding tree used to code all transcripts. These measures not only allowed the research team to identify salient findings that emerged from the data but also minimized the researcher’s potential biases (Figure 1). The coding tree was constantly revised and updated based on team discussions until agreement was achieved.

**Figure 2.**
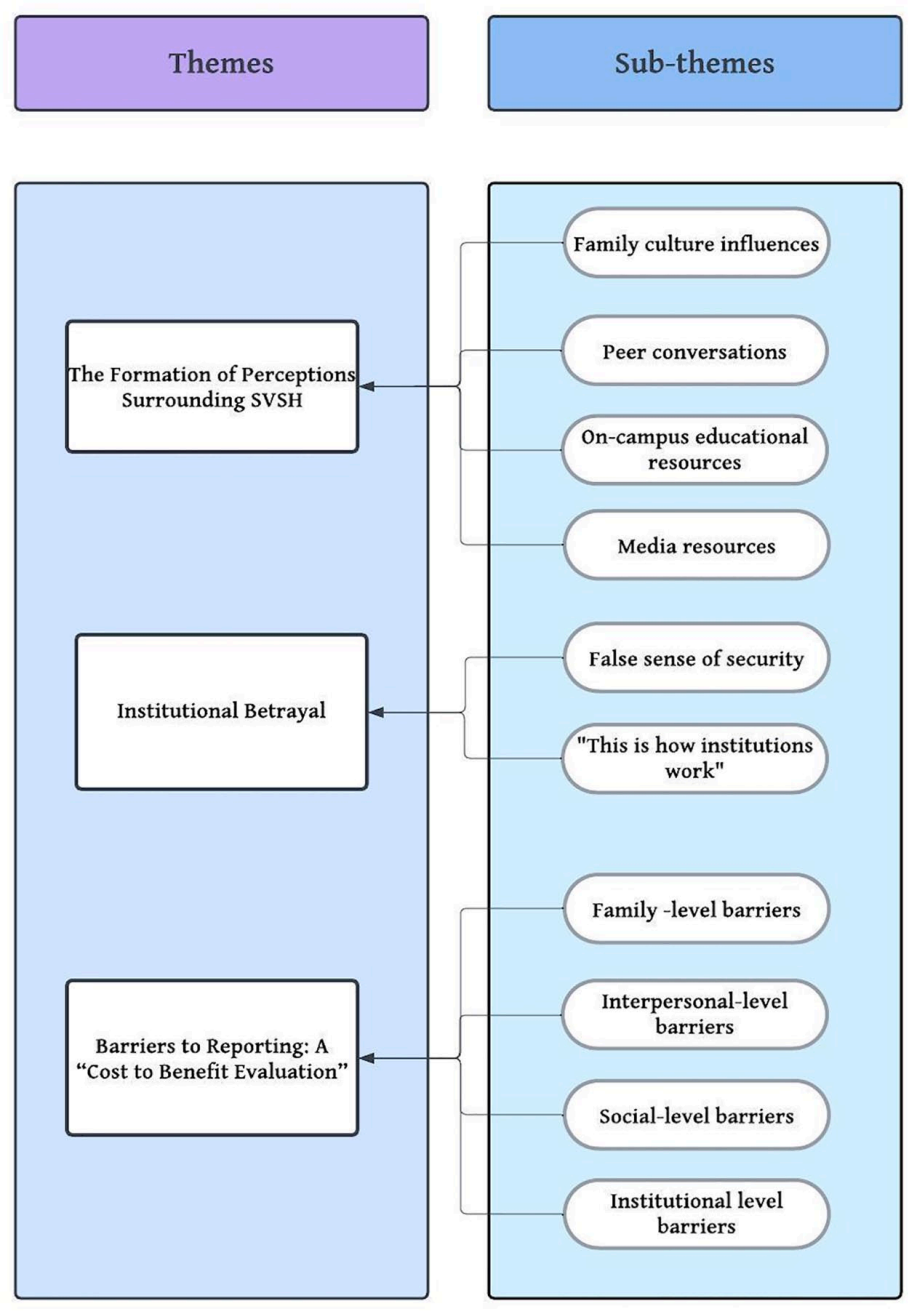
Themes and subthemes.

The finalized coding schema included three main themes and ten subthemes (Figure 2). Each transcript was cross-coded by two coders and discussed in depth among the research team to achieve consensus when disagreement on codes arose. The diversity of the research team’s academic backgrounds, with areas such as feminist studies, global studies, anthropology, public health, and social welfare, fortified the methodological approach through investigator triangulation.

## Results

### Participant Demographics

A total of 23 participants took part in the current study, all of whom self-identified as Asian and the largest proportion of participants were from UCLA (10/23, 43%). The majority (13/23, 57%) of participants identified as cisgender women, with cisgender men accounting for 39% (9/23) of the sample. About two-thirds indicated their sexual orientation was heterosexual/straight (15/23, 65%). Four participants identified as student-athletes (4/23, 17%). Members of the Greek community accounted for 17% (4/23) of participants (Table 1).

**Table 1.** Characteristics of the 23 Students Who Participated in IDIs.

Demographics	*N*/mean	%
Campus		
UCSB	5	22
UCSD	8	35
UCLA	10	43
Student status		
Undergraduate	19	83
Graduate	4	17
Mean age (range)	20.67 (18–28)	
Gender identity		
Cisgender female	13	57
Cisgender male	9	39
Agender	1	4
Sexual orientation		
Heterosexual/straight	15	65
Homosexual/lesbian/gay	2	9
Bisexual	1	4
Mostly heterosexual/straight but somewhat attracted to people of the same sex	1	4
Pansexual	3	13
Asexual	1	4
Involvement in athletics and Greek life		
Student-athletes	4	17
Fraternity or sorority member	4	17

*Note.* IDIs = in-depth interviews; UCLA = UC Los Angeles; UCSB = UC Santa Barbara; UCSD = UC San Diego.

### The Formation of Perceptions Surrounding SVSH

The formation of perceptions concerning SVSH among Asian students delves into multifaceted contributing factors. Our analysis explained three pivotal aspects: the influence of family culture, the consequential impact of peers, and the role played by on-campus educational resources alongside the effect of media resources. Within the subtheme of family culture, codes like “family being the least helpful” and “sex being a taboo in Asian families” were included. Peer interactions, as reflected in codes such as “using peers as the main educational sources,” shed light on their influential role. The impact of media resources became evident through codes like “had to turn to self-educating using online resources,” highlighting the reliance on alternative avenues. Furthermore, the codes related to college resources/training, such as “training being triggering and not trauma-sensitive” and “training was not relatable,” underscored the nuanced challenges within college environments.

#### Family Culture Influences

Asian college students expressed feeling more comfortable talking about sex and SVSH with friends or in public settings (e.g., classrooms, training, and organization events) as opposed to in their homes and with their parents or other family members. The participants attributed this discomfort of talking about such topics with families to the stigma surrounding sex and their perception that their parents had strong attitudes about the inappropriateness of openly discussing sexual matters:

They [the participant’s parents] don’t talk about it at all. I’ve never had any of these conversations with my parents or anyone related. I have spoken about it with my best friends at school, and (. . .) I had a lot of my information [from] sex ed.

Some students attributed this discomfort to the perceived stereotypical Asian culture that views sex as a “taboo” topic, a notion that has been supported by previous research (Kim, 2009). Anticipating negative reactions from their parents, most students said they often refrained from such conversations related to sex:

[There is] not so much [conversation in my] family (. . .) I feel like my parents are very traditional (. . .). It just doesn’t come up usually and I feel like they don’t value the topic as much as my peers do. I guess it’s just their own beliefs and values growing up.

While the lack of sex-related conversation at home was common across genders, Asian women participants discussed how conversations regarding SVSH with their parents primarily revolved around self-protection and avoiding “high-risk” situations, such as parties where alcohol is available. These discussions were often gendered, focusing on teaching females to “reduce the risk” of sex or violence by “acting or dressing safely.” This contrasts with parents taking an approach of talking to their children about how relationships should be gender equitable and mutually respectful. Overall, participants felt that their parents did not guide them on how to handle unsafe circumstances, including incidents of SVSH. An undergraduate who majored in humanities mentioned:

[Conversation with my family about sex] was more of an unspoken (. . .) like you need to be careful or you need to, as a woman, it’s your job not to act a certain way or look a certain way.

Another undergraduate student who majored in psychology also mentioned the gendered conversation at home:

No, that [SVSH] wasn’t really something we talked about [at home]. I mean, for the most part it was kind of like [my parents] would warn me to be wary of strange men, that kind of thing (. . .) otherwise it was more of just telling me to avoid it, but we didn’t really talk about it in detail.

Student participants described how the unique cultural aspects of family dialogs within the Asian community reinforced their beliefs that victims of SVSH bear responsibility for their experiences. Many felt that limited communication with their parents hindered discussions about sensitive but important issues such as the various forms of violence, including relationship violence, sexual harassment, stalking, and rape. Likewise, topics like safe sex practices and ways to seek support went unaddressed between Asian parents and their children. As a result, Asian students felt this contributed to their limited understanding of SVSH.

#### Peer Conversations

Participants highlighted informal conversations regarding sex and SVSH among friend groups as their primary source of learning about SVSH. Those conversations generally happened when an incident of SVSH occurred among their friend groups or when a friend utilized SVSH-related services:

Some incidences happened with people that were close to me (. . .) where I kind of had a personal learning experience with how it [SVSH] happens and how it works.

It was just in conversations with friends, and just discussing like the experiences that we’ve had and how that makes us feel.

These types of conversations showed gendered perspectives, where awareness and conversations surrounding SVSH were present among female-identified Asian students and lacking among their male-identified counterparts. Male participants expressed that the discussion of SVSH was avoided among their male peers due to stigma and lack of awareness. A few male participants discussed the topic of SVSH as uncommon and not taken seriously:

(. . .) Because rape culture isn’t just the incident, it’s the language we use to describe it. It’s the jokes that we tell them the things that we think are acceptable to joke about.

#### On-Campus Educational Resources

Most participants reported that they first learned about SVSH during college orientation, through campus safety training, or during academic classes. However, many of them expressed negative attitudes toward the training received through these formats, saying they felt the training was inadequate, unrelatable, and not trauma informed. Some described the SVSH education they received as “technical,” “unrelatable,” and “patronizing.” They also noted how the sessions were perceived as targeting male students while leaving the female students feeling that their voices and opinions were excluded:

I think that one of the big issues with these prevention workshops is that Title IX has a very top-down approach [about] how they talk to students (. . .) their workshops are super generalized and unrelatable and target only men, then it makes the woman in the room feel completely out of the picture.

Some students indicated that the school training was the first time they realized they had experienced SVSH, which subsequently led them to identify themselves as victims or survivors. One participant expressed that the sudden acknowledgment was not followed by any support from the training and resulted in a panic attack in public:

[The sexual violence prevention training] was a really traumatic experience for me to suddenly realize in the middle of a 500-person lecture that I had been sexually assaulted and suddenly had a panic attack in the middle of the auditorium. So that was, for me, very notably a terrible way of approaching sexual assault prevention [by] just shoving a bunch of freshmen into large lecture hall.

Asian international students narrated how they were confused as a result of limited provision of initial information about Title IX, cultural differences, unfamiliarity with the U.S. legal system, and different institutional practices. The terminology used in the training on the UC campuses often overlooked the potential challenges these international students might encounter when trying to understand SVSH concepts and vocabulary. This failure can be traced back to language and cultural barriers and the fact that educational institutions in different countries may have varying approaches to addressing issues related to gender discrimination and sexual misconduct. Title IX training introduced new concepts and procedures to many Asian international students. Participants explained how most of these new ideas differed from those they were introduced to in their home countries. Some students emphasized the struggles they encountered in understanding the complex notion of SVSH since terms are exclusively explained in English and most Asian international students said they had never previously been exposed to “any of the terms” before coming to the U.S. One graduate student stressed the necessity of providing international students with background information upfront:

Actually, no [the training is not effective] for us international students. We have no idea what Title IX is at first and they basically don’t give us any background information.

#### Media Resources

Media, encompassing a wide range of formats (e.g., news, social media, and television shows), was another common source of SVSH education for Asian college students. Students referred to the internet to learn more about SVSH by actively searching for media related to SVSH cases. Students also expressed that one of the main ways they gained SVSH knowledge, including how to identify red flags and self-care techniques, was through online media platforms, such as Twitter and Instagram:

[I learned the types of SVSH] Mainly online. Like honestly [on] Twitter there’s a lot of people if you branch out of your little bubble (. . .) I know some people do threads on Twitter about like what to watch out for, like red flags or like Self-Care 101.

### Institutional Betrayal

As discussed by Smidt and Freyd (2018), institutional betrayal manifests in many forms, including failure to prevent incidents, lack of supportive response to survivors, and cover-up of the administrators’ wrongdoing. This study’s findings reflected instances of “apparent isolation,” encompassing both acts of omission and commission (Smith & Freyd, 2014). The collected interview data revealed two primary subthemes: the illusion of campus safety and the acceptance of institutional norms. The analysis uncovered students’ misperception of on-campus safety with focus codes such as “assuming campus is safe” and “assuming college students know better but it’s not.” There is also a prevailing sense of institutional indifference toward individual concerns, indicated by focus codes like “perceiving students are not looked at as individuals by the institution.”

#### “This is How Institutions Work.”

Many Asian students expressed concern about their university’s response, or lack thereof, to SVSH. Participants felt that their school’s administration often disregarded cases of SVSH reports to protect the school’s reputation. More specifically, some students described how their institution prioritized actions that benefited the school, over those that would protect the student, likening the experience to a “marginal cost” evaluation. The perceived dehumanization of survivors of SVSH was believed to be part of “the institution’s job to protect the institution” and was thought to contribute to many students’ distrust in the university and its responses to SVSH:

I think in general the school is looking out for their greater being. . . and [students are] being treated like a client in some sort of business scheme or some sort of costs (. . .) [survivors are] being looked at as how much money are you costing the school.

Although Title IX offices are intended to remain neutral until perpetrators are proven guilty, many participants felt this approach conveyed the message that the Title IX officers “work with the perpetrator” instead of prioritizing victims’ or survivors’ well-being and safety. While students recognized the legal necessity of Title IX’s “innocent until proven guilty” approach, they expressed feelings of fear surrounding the potential judgment and invalidation (of their experiences) after reporting to Title IX. Students expressed a preconceived notion that the system would not protect them and would therefore simply “brush off” the report without any accountability placed upon the perpetrator:

[The participant’s campus has] not that great of a reputation for [on-campus resources] being responsible enough and taking accountability and actually making sure that there’s consequences for perpetrators of sexual violence and that survivors are protected. So, some people I’ve talked to feel that going to Title IX or going to UCPD [UC Police Department] would be a waste of time that would just re-trigger them.

Student participants shared strong feelings that establishing a safe environment for survivors is critical. However, most participants said they thought campus resources and university administrators had failed to achieve this objective. Asian students discussed how the university and the larger social system both contribute to silencing the survivors:

It is important that people are freer to speak out [about] their very traumatic experiences and see healing through that (. . .). I think that the institutions and society have both gotten better, but it’s still a very extremely vulnerable place to be in.

[Talking about a specific SVSH case on campus], the university [was] not acting adequately. I fully do believe that the university should have gotten rid of [the perpetrator] a long time before and there was a lot of neglect and trying to hide things in that case as well.

#### False Sense of Security

Another key theme related to institutional betrayal that emerged from the data was the phenomenon of a “false sense of security,” which led participants to believe their institution was safe and free from SVSH before enrolling as students. Participants felt this was a result of the university’s lack of effective training about SVSH risks and overall underreporting of SVSH cases. This “false sense of security” was a concept mentioned by students repeatedly when asked about the prevalence of SVSH on campus. Asian college students mentioned that people on campus tended to conflate spaces of higher education with safety, assuming their experience on campus would not put them in danger. On the contrary, students had an overall feeling that their peers were not provided with a proper understanding of how often SVSH occurs on their campus.

This false sense of security can be viewed from two angles: how the public views college students and how college students view themselves as a group. One student explicitly mentioned this concept when discussing consent and general assumptions about college students:

You would think that because we’re college students we’re highly aware of the issues going on and we’re smarter than that. But I think it tends to not be the case all the time. I think it’s this false sense of security knowing that college students have that high education and self-awareness.

Another participant described this concept and applied it to college students themselves:

I feel like [students] just assume that everybody else knows what to do. I mean, I used to be that way but when you meet your fair share of different people, you need to realize that not everybody is like you, not everybody’s nice, not everybody’s decent, not everybody is not an abuser.

Overall, female Asian participants tended to estimate higher rates of on-campus SVSH prevalence than their male counterparts. However, almost all participants, regardless of their gender, felt cases of SVSH were underreported. Some students mentioned how the dissonance between the perceived safety and the true risk for SVSH derives from the lack of communication from the university and the overall underreporting of SVSH cases. Some participants described the campus environment as “toxic,” and said that survivors’ voices are often “brushed off.”

### Barriers to Reporting: A “Cost to Benefit Evaluation”

The examination of barriers to reporting incidents unveiled layered obstacles across four levels: family, interpersonal, social, and institutional. At the family level, insufficient familial support hindered reporting. Interpersonally, fear of social repercussions from peers acted as a major block of help-seeking behaviors. In addition, negative perceptions acquired from media sources reinforced hesitancy to report SVSH incidents, and the institutional barriers indicated by focus codes such as “lack of trustworthy and accessible resources” add to the equation. These multi-dimensional barriers highlighted the complexity individuals face when evaluating the costs and benefits of reporting, contributing to the reluctance to seek support.

#### Family-Level Barriers

Related to how SVSH is a taboo topic in many Asian families, students expressed concern about their family members’ reaction to their experience, believing they may not receive the support they need. One student mentioned that this is because of the Asian culture of collectivism and fear of bringing shame to the family, referring to the societal values and norms of Asian culture that prioritize group harmony, family cohesion, and collective well-being over individual needs and desires:

In Asian culture, we’re really like a collaborative. So, everything that we do is reflected on family. And let’s say if you got into this accident, or you got into this sexual assault situation, then your whole family, like everyone’s just going to look at you in the way that you’re a bad person. So, you don’t want that to be you, and so that’s normally why you choose not to come out, because you don’t want to first dishonor your parents.

Sometimes parents would even blame the survivors for disobeying or dishonoring the family, which exacerbated the situation:

When you go back to your parents (after the SVSH incident), they look down on you, they said that “I”ve taught you this before, why would you do that?’

#### Interpersonal-Level Barriers

While peers were identified as a primary source of education about SVSH, participants expressed doubts about their peers’ ability to appropriately respond to disclosures of such incidents. Many Asian students recounted experiences where sharing their stories with peers led to ineffective responses, with peers often “freezing up” and feeling unequipped to handle the situation.

Participants also noted a reluctance among peers to support survivors, especially in cases where survivors opted to report the incident. This hesitance was attributed to fears of becoming entangled in legal proceedings, including potential involvement with school administration and the prospect of testifying in court, which was thought to be overly burdensome. A significant concern for Asian students was the potential breach of confidentiality by their peers. The fear that intimate details of their experiences might be shared and spread across campus, leading to rumors, was a prevalent worry. Participants highlighted that such breaches could result in “social backlash,” with survivors facing judgment and stigmatization within their peer groups:

The girls [survivors] can be called like a snitch (. . .) like guys are not gonna want to be friends with them anymore because they’re gonna have to be super, super cautious around the girls.

Several students noted that their male peers frequently labeled female students who report incidents of SVSH as “being overreactive,” which further contributes to the reluctance of survivors to come forward and report:

[After a girl reported an incident happened in a frat party], guys in that fraternity were just like “Oh, like it wasn’t even a big deal. Look, what are you talking about? She’s overreacting,” and obviously whenever she sees those guys on campus it’s gonna make her feel uncomfortable and so that’s going to scare girls away from coming out and telling their story.

Even for students who had not personally experienced SVSH, the negative stories largely increased their mistrust toward the campus resources (e.g., Title IX, campus police) and the fear of being socially excluded in their peer circle after reporting.

#### Social-Level Barriers

Many students were influenced by media that places blame on victim-survivors and “glorifies” or acquits perpetrators. This was said to be especially impactful when legal cases go public (e.g., Christine Blasey Ford, the survivor of the Brett Kavanaugh case received death threats while Kavanaugh went unpunished) (Hayes & Cummings, 2018; Mak, 2018; Bowden, 2018). The negative discourses surrounding reporting and seeking support services made participants internalize the belief that these systems would not be supportive, should they experience SVSH. Students felt that they would put themselves in danger of public criticism, similar to the cases reported on the news:

Sometimes [survivors] feel reluctant [to report] because of what they’ve seen in the media (. . .). Students may see that and internalize that in the same way (. . .) if they bring up a complaint of sexual harassment or rape then they receive threats (. . .) I think it just brings up huge discouragement to people who experience that (SVSH) themselves.

Asian international students were perceived to be particularly at risk since they tend to be more “hushed” in many social situations and the fact that their voices are often underrepresented in campus discussions of SVSH, let alone in the general public.

#### Institutional-Level Barriers

Students’ perception of school staff (e.g., Title IX personnel and departmental administrators), many of whom survivors may not have known personally prior to reporting, was also a consideration that participants felt students weigh when deciding whether to report. Participants felt that their institutions’ staff members, especially Title IX staff, had a reputation for being intimidating and not utilizing trauma-informed practices. Asian students perceived the aspect of reporting in which staff members must interview survivors about their experience of SVSH as “accusatory”:

They kept calling me in to all these different meetings and things, like “identify the person” (. . .) and then no one followed up with me (. . .) no one asked me like if I was okay.

The reporting and investigation processes were perceived as extremely lengthy, re-traumatizing, and lacking tangible results in terms of accountability for the perpetrator of violence. One participant told a story of a reported sexual assault incident, in which the alleged perpetrator was still “staying where he is”:

That guy’s not getting dropped from his fraternity. He’s not going to [be] expelled from the school. He’s staying just where he is.

Students also shared that lack of university accountability, even after an incident was reported, outweighs the benefit of reporting. With the effort required for the long and tiring process of reporting, some students felt it was not worth it to report:

Especially when the process is so complicated and so lengthy that people just give up on getting justice. Ultimately, I think it’s a lack of support and a lack of feeling like they’re supported.

With the negative discourses shared by the participants’ peers about cases being underreported and the ineffective services provided for reported cases, students expressed concerns with on-campus services, believing that there must be “something wrong with the system, or else people would start doing it [reporting] more.” One participant summarized the impact of the discourse as:

Survivors can be reluctant [to report] because they think maybe it’s because they [on-campus services] don’t see us [survivors], if they [students] keep hearing that it’s not effective or it’s not being successful.

Overall, participants expressed concerns about negative consequences from parents, peers, school staff, and the public interacting with feelings of self-blaming from survivors and the lack of accountability for perpetrators of SVSH. This, students felt, led to survivors engaging in a “cost–benefit analysis” of whether to report their experience of SVSH, largely resulting in survivors being discouraged and afraid to do so:

What’s the benefit of me reporting this [SVSH]? Right? If I don’t report it, I save the time, I save my friends through this misery. I won’t get shamed upon[me] by these friends who care about them [the perpetrator]. And maybe I can figure it out on my own.

At least if you keep it [SVSH] a secret or to yourself, then it’s just you and your truth. If you put that out into the world, people can start to criticize you or your actions, which sucks because it [SVSH] isn’t your fault.

## Discussion

The issue of SVSH on campuses is a matter of ongoing concern; yet, the discourse often predominantly focuses on racial majority students, leaving the experiences of various minority groups, including Asian students, underexplored (Boykins et al., 2010; McMahon & Seabrook, 2020). This bias may stem from the fact that minority groups’ experiences and responses to SVSH could be influenced by specific sociocultural factors that are less understood. Among these underrepresented groups, Asian students constitute a significant segment in higher education, and their unique cultural and societal perspectives might shape their understanding and reactions to SVSH differently than their counterparts from other racial backgrounds. By exploring the experiences of the relatively unexamined Asian student population, our study provided evidence of this population’s experience with a unique cultural lens that affects Asian students’ SVSH perception and help-seeking behaviors.

The findings highlighted that certain Asian family norms regarded sexual topics as sensitive, causing a lack of conversation about SVSH and leading to gender-specific prejudice that prioritizes female self-protection. The study also uncovered a sentiment of institutional betrayal in universities, with Asian students perceiving a lack of support, inadequate SVSH training, and a tendency for institutions to protect their reputation over students’ well-being. These factors contributed to an overall environment where students felt discouraged and fearful to report SVSH incidents, often engaging in a cost–benefit analysis which leaned toward silence due to potential negative consequences from family, peers, and institutional authorities.

Consistent with previous research on SVSH in college campus environments, our study demonstrated how factors across different socio-ecological levels shaped Asian students; perceptions of and responses to SVSH. These factors included the following: (a) individual-level perceptions of self-blame, guilt, and fear related to SVSH victimization (Gilligan & Akhtar, 2006; Kenny & McEachern, 2000; Koo et al., 2012, 2015; Okazaki, 2002; (b) interpersonal dynamics with family and friends that were negative or unsupportive (Kim & Ward, 2007; Kim, 2009; Liang et al., 2008; Malik et al., 2020; Morgan et al., 2010; Trinh & Kim, 2021); (c) complicated and often distrusted institutional procedures encompassing Title IX, campus police, and university administration (Goldman, 2019; Gómez, 2022; Monteith et al., 2016; Smith & Freyd, 2013); and (d) societal norms that perpetuated negative discourse surrounding SVSH (Brubaker et al., 2017; Daigle et al., 2018; Holland & Cortina, 2017; Postel, 2015, 2020; Scholl et al., 2021).

Particularly, the results showed how Asian students felt a sense of institutional betrayal toward on-campus resources and their university’s reporting system. Students tended to perceive the goal of on-campus service staff as protecting the institution, rather than ensuring the safety and well-being of the survivors. This mistrust was exacerbated by the “innocent until proven guilty” principle of services, specifically Title IX. While the initial neutral stance is a courtroom standard, it is most appropriate for campus services to implement survivor-centered and trauma-informed services simultaneously throughout the investigation process to counter the perception among students, especially student survivors. Campuses can also enhance clear communication with students regarding their rights, available resources, and the investigation process. Providing information in multiple languages will help ensure that all students can fully understand their options and available support.

Our study built upon the findings of “UC Speaks Up,” the parent study from which our data originated. While “UC Speaks Up” explored various aspects such as undergraduate students’ perceptions and recommendations on SVSH (Bloom et al., 2022), the viewpoints of graduate students (Bloom et al., 2021), the concept of institutional betrayal among the general student body (Bloom et al., 2023), and the importance of confidential services (Mitra et al., 2021), our research delved further into understanding the impact of race/ethnicity on perceptions and responses to SVSH, by focusing on Asian students. For Asian students attending public universities in Southern California, particularly those from tightly knit Asian communities, a significant barrier to reporting SVSH is the cultural focus on upholding “family honor,” which added an extra obstacle. The lack of family support deterred Asian students from discussing SVSH with their parents or seeking help from family members post-SVSH incident. Concerns about dishonoring the family, rooted in misogynistic beliefs, and the stigma placed on victims of SVSH, led many to silence. Prioritizing collective honor over individual experiences, Asian students heavily relied on their peers and media for information and support related to SVSH. However, fear of inadequate support and societal judgment made students hesitant to share personal experiences and seek help when they needed it.

Moreover, the distinct experiences of Asian international students accentuated their encounters with additional hurdles when it came to seeking support. Those challenges included limited awareness about the available services, unfamiliarity with both on-campus support mechanisms and the Title IX process, and a dearth of tailored training resources to address their specific requirements. Notably, the unfamiliarity with the U.S. legal system and language barriers, as highlighted in the preceding results, particularly pertained to international students. Given the prevalence of these issues among participants from the UC system, where approximately 78% of international students originate from Asian countries (University of California, 2023), it becomes imperative to adequately tailor the prevention and intervention services to their needs. This underscored the importance of future research in comprehending how their unique identities and social standings shape their interactions with SVSH concerns.

### Strengths and Limitations

This study is one of the few qualitative examinations of Asian college students’ perceptions and experiences of on-campus sexual violence, survivor support services, and reporting choices. It provides a novel perspective of the interaction between Asian cultural factors and campus climates, and how this interaction impacts Asian college students’ experiences. The study’s sample was intentionally drawn from a population of students from diverse backgrounds, allowing for the examination of various perspectives and elevated voices from historically underrepresented subgroups.

However, we acknowledge that this study is not exempt from limitations. First, the interview guide was designed for the general student population, therefore, not specifically asking questions related to Asian cultures and Asian identities. The information we acquired through the interviews could have been enriched with more guiding questions targeting this population, however, not directly querying about Asian or cultural factors highlights the salience of this study’s results for the participants. In addition, we acknowledge the term “Asians” as a wide umbrella for over 50 distinct ethnicities, each with unique cultural, linguistic, and socioeconomic characteristics. While the research indiscriminately analyzed the experiences of all Asian students, ranging from international to Asian American, this broad approach risks overgeneralizing their narratives.

Our study is also subject to many limitations inherent to qualitative research. For instance, our findings might be influenced by researcher biases and may not be generalizable to the larger UC population or other college and university campuses. Our study also lacks an assessment of interrater reliability which may affect the consistency and objectivity of our findings. Furthermore, the data did not distinguish between various on-campus resources, inhibiting a nuanced understanding of attitudes toward services like Title IX, CARE, UCPD, etc. For more comprehensive insights, future studies should investigate the experiences of Asian college students in specific ethnic subgroups and evaluate how their unique cultural contexts interact with different campus support systems.

### Implications

With the on-campus services often not fully aware of Asian students’ experience of needs and barriers, we call for more culturally informed practices to increase the trust in the on-campus SVSH resources among Asian students. The practice reformation may entail tailoring survivor support services to Asian students (e.g., providing cultural training and hiring staff from diverse backgrounds), providing multilingual SVSH educational materials and training, and promoting confidential services without filing formal reports, which would reduce the risk of social repercussions. In addition, to better understand and accommodate the unique cultural background of racial minority students, colleges can facilitate peer support groups so that the shared contexts would be taken into consideration when providing support.

Overall, this study points to multiple factors contributing to Asian college students’ perception of SVSH and perceived barriers to reporting. After SVSH incidents, Asian student survivors often weigh these perceptions and assess the potential social cost of reporting their SVSH experiences. Their personal, culturally informed analysis leads to their decision to establish a solution that best serves their needs, even if that means not pursuing formal reporting processes or seeking external survivor support resources, which is well reflected in the following quote:

The negative consequences from reporting it [sexual assault] that make them [victims] feel reluctant to share it. Victims can be reluctant. . .because they [institutions] don’t see us [victims].

## Notes

1. Asians residing in the U.S. represent a population from more than 50 different ethnicities and 100 different languages. Asians are diverse in characteristics such as country of origin, religion, generational status (e.g., U.S.-born versus foreign-born), duration of residence and citizenship status in the U.S., and socioeconomic indicators (Nadia et al., 2010).
